# Best strategies for patient education about anticoagulation with warfarin: a systematic review

**DOI:** 10.1186/1472-6963-8-40

**Published:** 2008-02-14

**Authors:** James L Wofford, Megan D Wells, Sonal Singh

**Affiliations:** 1 Department of Internal Medicine, Wake Forest University School of Medicine, Winston-Salem, North Carolina, USA

## Abstract

**Background:**

Patient education is an essential component in quality management of the anticoagulated patient. Because it is time consuming for clinicians and overwhelming for patients, education of the anticoagulated patient is often neglected. We surveyed the medical literature in order to identify the best patient education strategies.

**Methods:**

Study Selection: Two reviewers independently searched the MEDLINE and Google Scholar databases (last search March 2007) using the terms "warfarin" or "anticoagulation", and "patient education". The initial search identified 206 citations, A total of 166 citations were excluded because patients were of pediatric age (4), the article was not related to patient education (48), did not contain original data or inadequate program description (141), was focused solely on patient self-testing (1), was a duplicate citation (3), the article was judged otherwise irrelevant (44), or no abstract was available (25).

Data Extraction: Clinical setting, study design, group size, content source, time and personnel involved, educational strategy and domains, measures of knowledge retention.

**Results:**

Data Synthesis: A total of 32 articles were ultimately used for data extraction. Thirteen articles adequately described features of the educational strategy. Five programs used a nurse or pharmacist, 4 used a physician, and 2 studies used other personnel/vehicles (lay educators (1), videotapes (1)). The duration of the educational intervention ranged from 1 to 10 sessions. Patient group size most often averaged 3 to 5 patients but ranged from as low as 1 patient to as much as 11 patients. Although 12 articles offered information about education content, the wording and lack of detail in the description made it too difficult to accurately assign categories of education topics and to compare articles with one another. For the 17 articles that reported measures of patient knowledge, 5 of the 17 sites where the surveys were administered were located in anticoagulation clinics/centers. The number of questions ranged from as few as 4 to as many as 28, and questions were most often of multiple choice format. Three were self-administered, and 2 were completed over the telephone. Two reports described instruments along with formal testing of the validity and reliability of the instrument.

**Conclusion:**

Published reports of patient education related to warfarin anticoagulation vary greatly in strategy, content, and patient testing. Prioritizing the educational domains, standardizing the educational content, and delivering the content more efficiently will be necessary to improve the quality of anticoagulation with warfarin.

## Background

Warfarin is a dangerous outpatient medication, by anyone's estimation. It is the second most common cause of adverse drug events in emergency rooms, and the overall risk of major bleeding averages 7–8% per year [1,2]. Despite the risk, well-established indications for warfarin are increasing in prevalence with aging of the population [3,4], and new indications for warfarin are regularly recommended [5,6]. As a result, the proportion of elderly persons taking warfarin has risen to as high as 7% [7].

Increasing a patient's understanding about warfarin is a logical goal. Prior knowledge about warfarin has been associated with a decreased risk of bleeding [8]. Written and verbal information has been shown to improve control of the level of anticoagulation [9]. While past studies suggest that patient education may be associated with better clinical outcomes, doubts remain about the effectiveness of patient education strategies [10-12]. As a result, systematic patient education regarding long-term warfarin is not universally implemented.

Our objectives were to (1) identify the published strategies (duration, timing, personnel requirements, content domains) for patient education regarding warfarin anticoagulation and (2) identify published instruments for measuring patient knowledge.

## Methods

In March 2007, we searched MEDLINE using the MESH terms ("warfarin" or "anticoagulation") AND "patient education". We limited our search to articles published in the English language. We used the related articles link in PubMed and searched the references of identified citations for additional original articles. Similar search terms were used to search Google Scholar. As warfarin is by far the most commonly used oral anticoagulant, we did not seek articles related to other oral anticoagulants.

We sought articles that (a) were original research studies or descriptions of patient education programs that included information on the educational content and strategy related to anticoagulation with warfarin, or (b) contained instruments that measured patient knowledge. Exclusion criteria included studies conducted in pediatric populations, unrelated to patient education, lacking original data or an adequate program description, and those in which the educational effort was focused solely on patient self-testing. Because citations might be excluded for multiple reasons, we used this above mentioned sequence for excluding citations.

An initial search identified 206 citations. Two reviewers (JLW, MDW) reviewed titles and available abstracts to determine relevance to the stated objectives of identifying (1) the optimal educational content and delivery (duration, timing, personnel requirements), and (2) the optimal strategies for measuring patient knowledge. Full text articles were retrieved for citations that met our inclusion criteria and for those where inclusion/exclusion criteria were not determinable by the title and abstract. Two other citations were encountered during the process of reviewing articles that were deemed eligible, raising the number of eligible articles to 208.

**Figure 1:**
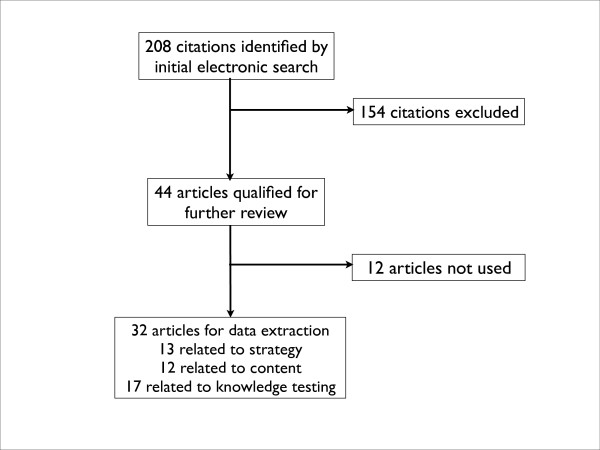
Search strategy for studies and programs related to patient education about warfarin anticoagulation.

A total of 154 citations were initially excluded because patients were of pediatric age (1.9%, 4), the article was not related to patient education (23.1%, 48), did not contain original data or inadequate program description (18.8%, 39), was focused solely on patient self-testing (1), was a duplicate citation (1.4%, 3), or the article was judged otherwise irrelevant (16.8%, 35), or no abstract was available (11.5%, 24) (Figure 1).

After exclusions, a total of 44 articles qualified for further review. Upon further review, an additional 12 articles were excluded because of inadequate program description, ultimately leaving a total of 32 articles for data extraction (Figure 1). We extracted data on clinical setting, study design, group size, content source, time and personnel involved; and created summary tables. Two reviewers (MDW, JLW) identified the educational topics covered in these reports. Among studies that tested patient knowledge, we extracted information on setting and study population, number and type of questions, and method of administration.

## Results

Thirteen articles had a description of the research methods or program that was adequate and consistent with our objectives of identifying the duration, timing and setting, and personnel requirements of the educational program (Table 1) [13-25]. Five programs used a nurse or pharmacist (45%), four used a physician, and two studies used other personnel/vehicles (lay educators (1), videotapes (1)). The duration of the educational intervention ranged from one to ten sessions. Patient group size most often averaged three to five patients but ranged from as low as one patient to as much as eleven patients. While the majority of the educational efforts occurred in inpatient settings, most seemed relevant to contemporary outpatient settings.

**Table 1:** Patient Education Strategies Related to Warfarin and Anticoagulation

**Citation Location Study Design**	**Stated Goal**	**Group Size**	**Personnel involved**	**Strategy/Duration/Frequency**
Menendez-Jandula et al^13 ^2005 Barcelona, Spain RCT	To prove the value of self-management on INR control and clinical outcomes	5–8 patients and option of having family member present	Specially trained nurse	2 sessions of 2 hours on consecutive days Based on German model
Koertke et al^14 ^2005^1 ^Westphalia, Germany Program description	To describe the principles of a training course to learn INR self-management	Not more than 5 patients	Not stated	Welcome period Two phase (hospital, 6 months later) Average duration 3–4 hours (1.5 for theoretical and 1.5 for device handling)
Voller et al^15 ^2004^1 ^Westphalia, Germany Program description	To evaluate the effects of a training program on patient knowledge	2–5 patients	Not stated	Two half day sessions 2–7 days apart. Patient logbook
Khan et al^16 ^2004 Newcastle, U.K. RCT	To prove the value of education and self-monitoring on INR control and quality of life	2–3 patients	Led by physician	1 two hour educational session
Gadisseur et al^17 ^2003 Leiden, Netherlands RCT	To examine effects of self-management on quality of life	4–5 patients	Specialized teams of physicians and nurses	3 weekly sessions of 90–120 minutes
Singla et al^18 ^2003 Philadelphia, U.S. Cohort Survey	To examine effects of group education on knowledge	11 persons	Pharmacist or nurse	1 one hour session
Amruso^19 ^2003 Tampa, U.S. Program description	To examine effects of group education on knowledge	One-on-one	Chain pharmacy pharmacist	Ongoing monthly appointments
Beyth et al^20 ^2000 San Francisco, U.S. RCT	To prove the value of self-management	One-on-one	Lay educator	Specifically formatted workbook. Coaching on communication skills. Self monitoring
Morsdorf et al^21 ^1999 Saarland, Germany Program description	To examine the efficiency of patient training for self-management	4–6 patients	Single MD\Single instructor	4 theoretical and 2–6 practical sessions Video assisted demonstrations
Foss et al^22 ^1999 Denver, U.S. Program description	To describe the efficiencies of a high-volume anticoagulation clinic	Not more than 6 patients	Pharm D	1 hour slide presentation
Sawicki et al^23 ^1999 Dusseldorf, Germany RCT	To prove effect of self management on accuracy of control and quality of life	3–6 patients	Physicians and nurses	3 consecutive weekly teaching sessions of 60 to 90 minutes in duration
Stone et al^24 ^1989 Worcester, U.S. RCT	To examine the effect of videotape on knowledge	One-on-one	Nurse	15 minute videotape compared with 25 minute nurse lecture
Scalley et al^25 ^1979 San Antonio, U.S. Program description	To develop a program for patient education	One-on-one	Pharmacist or nurse	Average 30 minutes. Slide presentation and booklet. Checklist of learning objectives placed in patient's chart

Although twelve articles offered information about education content, the wording and lack of detail in the description made it too difficult to accurately assign categories of education topics and to compare articles with one another [2,11,12,15,19,22-24,26-29]. Nevertheless, we summarized the categories suggested by these studies and listed the potential topics for each category (Table 2).

**Table 2:** Topics for Education of the Anticoagulated Patient

**Category**	**Educational Topic**
Basics of anticoagulation	
	Description of the coagulation system
	Normal blood clotting compared with clotting of an anticoagulated patient
	Warfarin – mechanism
Risk-Benefit	
	Risk of bleeding versus – descriptive versus numerical
	Risk of clotting – descriptive versus numerical
	Complications of thromboemboli
Adherence	
	Color and strength of tablets
	What to do if dose missed
Accessing healthcare professionals	
	When to call the doctor
	When to seek emergency care
	Anticoagulation services
Diet	
	Basics of Vitamin K
	Specific foods
Lab monitoring	
	Basics of the INR
	Therapeutic INR range
	Most recent INR Result
	Interpretation of INR values
	Frequency of INR determination
Medication interactions	
	Antibiotics
	OTC medications
Self-Care	
	Injury management and contraindicated activities
	Signs of bleeding events (overdose)
	Signs of thromboembolic events (underdose)
	Management of minor bleeding events
	Medical alert bracelet
	Special situations – illness, travel, pregnancy, surgeries
	Endocarditis prophylaxis
Self-testing	
	Dose adjustment
	Home coagulometry
	Diary/quality control record keeping

Relevant to our objective of identifying measures of patient knowledge, Table 3 shows the seventeen relevant citations [9,11,12,15,18,24,30-40]. Five of the seventeen sites where the surveys were administered were located in anticoagulation clinics/centers. The number of patients included in these studies ranged from as low as 22 to as high as 530. The number of questions ranged from as few as 4 to as many as 28 questions, and were most often of multiple choice format. Three were self-administered, and two were completed over the telephone. Two citations [12,32] described testing instruments along with formal testing of the validity and reliability of the instrument.

**Table 3:** Studies Testing Patient Knowledge Regarding Anticoagulation

**Citation**	**Setting/Study population**	**Questions – Number and Type**	**Administration**
Hu et al^30 ^2006	Large urban teaching hospital 100 mechanical valve patients	20, True-False	Scripted telephone survey Trained medical student
Zeolla et al^12 ^2006 OAK test	U.S., Recruited from 4 pharmacies and 2 clinics 122 volunteers	20, Multiple choice, Validity and reliability testing	Self administered Excluded illiterate patients 7th grade reading level
Roche-Nagle, Chambers^31 ^2006	Dublin teaching hospital anticoagulation clinic 150 consecutive patients	8, Specific answers	Standardized interview
Davis et al^11 ^2005	Two NYC anticoagulation clinics 52 patients	18, Multiple choice	Self administered Single visit Excluded low literacy patients
Briggs et al^32 ^2005 AKA test	Two Chicago inner city, pharmacist-managed anticoagulation clinics 60 patients	28, Multiple choice, Validity and reliability testing	Self administered Excluded illiterate patients 7th grade reading level
Voller et al^15 ^2004	Three German 3 teaching centers 76 patients	13, Multiple choice	Questions not available
Nadar et al^33 ^2003	3 U.K. teaching hospital anticoagulation clinics 180 patients who attended the clinic > 5 times	9, Short answer	Language concordance, personal interview
Tang et al^9 ^2003	1 Hong Kong anticoagulation clinic 56 patients months postdischarge	9, Dichotomous and open-ended	2 rehearsed pharmacy students Leading questions avoided Scoring details
Cheah et al^34 ^2003	U.S. teaching hospital center 50 inpatients	10, Open-ended	Telephone survey
Singla et al^18 ^2003	U.S. anticoagulation center 180 patients	4, Yes/No	Immediately after class
Wilson et al^35 ^2003	U.S. urban university hospital anticoagulation clinic 65 patients	20, Short answer	Investigator interview one week post discharge. Instrument not available
Barcellona et al^36 ^2002	Italian thrombosis center 216 patients taking warfarin for 6 months	6, Multiple choice	Self-administered
Waterman et al^37 ^2001	U.S. managed care organization 530 patients	11, True-False or short answer	Telephone-based interview at enrollment
Wyness et al^38 ^1990	U.S. university hospital vascular surgery unit 23 patients	Interview soliciting explanation	Before discharge, and 1 & 3 months after discharge Oral interview
Stone et al^24 ^1989	Hospital-based anticoagulation clinic 22 patients	18, True-False	Self-administered
Rankin^39 ^1979	University hospital cardiac rehabilitation unit 19 patients	14, Multiple choice	3–4 days later and 3 weeks later Investigator administered
Clark et al^40 ^1972	U.S. university hospital, 45 patients	15, Multiple choice	Self administered

## Discussion

Patient education has long been thought to be useful for patients receiving long-term anticoagulation. Proposals have been periodically issued suggesting the content of the educational task [2,23,41]. However, inadequate attention to health education principles and educational program design have more often been the problem than have issues of content [29,42]. Despite the practical value of making the patient as knowledgeable as possible, the best strategy for educating patients about anticoagulation is yet to be determined [10].

The variety of strategies shown in Table 1 likely reflect a varying amount of support and resources devoted to this patient education goal. Delegating these educational activities to midlevel practitioners, pharmacists, or designated nurses are strategies well supported by the our literature review. However, in any given clinical setting, local factors such reimbursement and available manpower may determine which health professional(s) is best responsible for managing a population of anticoagulated patients. The advent of warfarin self-monitoring with home coagulometers has sparked renewed interest in improving patient education related to anticoagulation [2,13]. Government-supported efforts in Germany and Netherlands now devote a significant level of time and manpower to this educational task [21,43]. However, most clinical settings in the U.S. and elsewhere, may not be able to match that level of support [15]. Because most anticoagulation management still takes place in the offices of clinicians [44,45], strategies to provide education should be relevant to all clinical settings.

We also found much variability in the content areas reported by educational programs, to the degree that we could not accurately categorize educational domains, let alone make fair comparisons among programs. Some issues (manifestations of bleeding, INR monitoring, etc) were a component of most educational programs, while other issues (Vitamin K, pill color) were present only in a few. Our inability to summarize published efforts likely reflects an underreporting of details rather than extreme variability among programs. Nevertheless, our table of potential educational topics (Table 2) reflects a daunting agenda.

The testing of patient knowledge regarding warfarin and anticoagulation used a variety of instruments. Only two of the sixteen instruments – the Oral Anticoagulation Knowledge (OAK) instrument and the Anticoagulation Knowledge Assessment (AKA) – have been subject to any formal evaluation. The Oral Anticoagulation Knowledge (OAK) investigators evaluated construct and content validity, test-retest reliability, and internal consistency reliability [12]. The Anticoagulation Knowledge Assessment (AKA) investigators used the Rasch model in order to examine validity, and item and person reliability [32]. Both the OAK and AKA are reported to be written at the 7th grade reading level, but neither instrument has been validated in other clinical settings. The best strategy for measuring patient knowledge would depend, in part, on the content of the educational program, but standardization of the testing effort should be a realistic goal.

The limitations of our study deserve acknowledgement. While our study reflected a variety of different strategies for all aspects of the educational process, it is probable that noteworthy and innovative patient education efforts may not be reflected in the medical literature. Second, in reviewing these reports, it is often difficult to separate the management strategy from the educational strategy.

Despite the variability in the content and strategies of educational programs, several important issues should drive future efforts at patient education, in our opinion. Educational programs should focus on topics essential for patient safety, such as what to do when INR is high, rather than the minute details of anticoagulation that overburden the patient. Second, these programs would best be implemented with measures of effectiveness and improvement in patient knowledge, adherence and outcomes using validated instruments. Lastly, educational programs should attempt to maximize office efficiency by delegating this task to physician extenders, nurses, pharmacists, or perhaps an office-based computer.

## Conclusion

Patient education is entering a new era where accountability in educational outcomes, interest in literacy/language barriers, and the importance of cost-effectiveness will influence the process of patient education. Prioritizing the educational content and using validated instruments for measuring the outcomes of patient education will be a necessary first step in improving anticoagulation outcomes. This systematic review should guide future efforts.

## Competing interests

The author(s) declare that they have no competing interests.

## Authors' contributions

Please see sample text in the instructions for authors.

## Pre-publication history

The pre-publication history for this paper can be accessed here:


